# Assessment of Antiretroviral Treatment Adherence among Children Attending Care at a Tertiary Hospital in Southeastern Nigeria

**DOI:** 10.1155/2017/3605850

**Published:** 2017-02-05

**Authors:** Cletus Akahara, Emeka Nwolisa, Kelechi Odinaka, Seline Okolo

**Affiliations:** ^1^Department of Paediatrics, Federal Medical Centre, Owerri, Imo State, Nigeria; ^2^Department of Paediatrics, University of Jos Teaching Hospital, Plateau State, Nigeria

## Abstract

*Background*. Adherence is the strongest predictor of successful treatment outcome among children infected with HIV. Our aim was to assess the antiretroviral drugs adherence status of HIV-infected children attending care at a tertiary hospital in Southeastern Nigeria.* Method*. The study involved a cross-sectional survey of 210 HIV-infected children attending care at a tertiary hospital in Southeastern Nigeria using self-report method of assessment. Optimal ART adherence is defined as patient taking not missing more than 1 dose of combined antiretroviral therapy medication in the preceding 2 weeks prior to the study.* Result*. A majority of the subjects 191 (91%) had good adherence. There was a significant relationship between adherence and patient educational level (*p* = 0.004), duration of treatment (*p* = 0.001), drug administrator (*p* = 0.005), and orphan status (*p* = 0.001). The motivating factor for adherence was “not falling sick as before” while stigma was the most discouraging factor.* Conclusion*. The adherence level in this study was good. Stigma was an important reason given by patient/caregivers for nonadherence. There is need for concerted effort in addressing this barrier to improve adherence and prevent the emergence of drug resistance and treatment failure.

## 1. Introduction

Human Immunodeficiency Virus (HIV) infection and the resultant Acquired Immunodeficiency Syndrome (AIDS) have remained of significant public health concern especially in sub-Saharan Africa where they still contribute significantly to childhood morbidity and mortality [1]. There are yet no curative drugs but the use of combined antiretroviral therapy (cART) has ensured better outcomes. cART consists of the use of mostly three antiretroviral agents and good adherence is the most important determinant of success and positive outcome [2].

With regard to antiretroviral therapy (ART), an adherence level of 95% or more is required in order to obtain successful outcome [3]. Suboptimal adherence can lead to incomplete viral suppression, emergence of resistant viral strains, and treatment failure. Paediatric adherence relies on the child and his/her caregiver and as such is especially challenging [4–10].

Given that adherence is the strongest predictor of successful treatment outcome [2], a clearer understanding of adherence barriers is very crucial in a bid to develop measures to support and sustain patient's adherence in the clinical care of HIV-infected children.

Several methods have been used to measure adherence and they include Therapeutic Drug Monitoring (TDM), Directly Observed Therapy (DOT), Pill counts, Medication Event Monitoring System (MEMS), and self-report.

In the self-report method, the patient or caregiver in the case of children gives information on how the drugs are taken and reports on missed doses. The method hinges on the client providing true and accurate information.

This study is set to assess the antiretroviral drugs adherence status as well as identify reasons influencing adherence of HIV-infected children attending the Paediatric HIV Disease Clinic of the Federal Medical Centre, Owerri, Imo State, Nigeria.

## 2. Method

The study was conducted at the Paediatric HIV Clinic of the Federal Medical Centre, Owerri, Imo State, Nigeria. The centre is one of the major HIV/AIDS treatment centres in the South East geographical region of Nigeria.

### 2.1. Ethical Approval

The Research and Ethical Committee of Federal Medical Centre, Owerri, gave ethical clearance for the study. Informed written consent was obtained from the caregivers and assent from children older than 10 years.

*Inclusion Criteria*

1. HIV-infected children aged 10–180 months
2. HIV-infected children on cART for at least 4 months
3. Informed consent from caregiver

*Exclusion Criteria*

1. Subjects above 15 years
2. HIV-infected children yet to commence cART
3. Subjects whose caregiver did not give informed consent

### 2.2. Study Duration

The study was carried out over a 4-month period (September to December 2013).

### 2.3. Data Collection

A pretested questionnaire was used for data collection. The information sought included the caregiver's related factors such as age, educational level, occupation, relationship to subject, and treatment duration. Also the patient's related factors like age, gender, education, orphan status, and missed doses were sought.

Adherence was determined using the self-reporting method. Adolescent patients and their parents/caregivers (for the younger subjects below 10 years of age) were asked to recall how many times they missed taking drugs as prescribed in the two weeks preceding the study. Patients were classified as having good adherence if they scored ≥95% (not missing more than 1 dose).

### 2.4. Data Analysis

The data from the questionnaire were sorted, coded, and entered into the computer and analyzed using the SPSS version 20.0, Chicago, Illinois, USA. The data was further subjected to descriptive and analytical statistics to generate frequencies and percentages. Chi square was used to test for association between two categorical variables.* p* value < 0.005 is considered to be statistically significant.

## 3. Results

A total of 210 patients who met the inclusion criteria were enrolled in the study. There were 108 males and 102 females giving a male: female ratio of 1.05 : 1.

The sociodemographic characteristic of patients studied is shown in Table 1. 136 (64.8%) of the studied patients were aged 12–119 months while 106 (50.5%) of the studied patients had been on cART between 12 and 35 months. 146 (69.5%) had their drugs administered by their biological parents. While 64 patients (30.5%) were single orphans and 22 (10.5%) were dual orphans.

191 (91%) had good adherence. Adherence in female patients was 92% while it was 90% in the male subjects. The difference though was not statistically significant (*p* = 0.554). There was a significant relationship between adherence and patient educational level (*p* = 0.004), duration of treatment (*p* = 0.001), drug administrator (*p* = 0.005), and orphan status (*p* = 0.001).

Table 2 shows the reasons for good adherence. The major reason for good adherence was the patient was not as sick as before.  Table 3 shows the reasons for poor adherence. These information pieces were given by the patient.

## 4. Discussion

The adherence rate among patients in this study was 91%. This corroborates the study of Iroha et al. [11] and Mukhtar-Yola et al. [12] but differed from the study of Ugwu and Eneh [6] in Port-Harcourt, Nigeria. These studies all used the self-reporting method in assessment of adherence. Whereas our study assessed adherence by recall over the previous two weeks, Ugwu and Eneh [6] assessed recall over a one-month period. The lower adherence levels reported by Ugwu and Eneh [6] may be associated with recall bias against a shorter period (two weeks) used in the present study.

The percentage of patients being adherent was observed to differ across age groups. This pattern was also observed by Zubayr et al. [13] and Iroha et al. [11]. The pattern of decreasing adherence with increasing age agrees with reports from Kampala [14], Ethiopia [15], and Cape Town [16] which documented a worsening ART nonadherence with increasing patient's age. This is natural as children do not like taking drugs every day of their life and they also do not like to appear different from their peers.

Despite sociocultural preference for male children by the Igbos of Southeastern Nigeria, our study observed that adherence was higher in females than males, although the difference was not significant. This observation corroborates earlier reports by Ugwu and Eneh [6] and Zubayr et al. [13] that found no significant association between the gender of the child and ART adherence. This implies that gender may not influence ART adherence.

Adherence was higher in patients whose biological parents administered the drugs. The relationship between drug administrator and adherence was statistically significant. This study finding is in tandem with the study of Zubayr et al. [13]. This may not be surprising as the parents were responsible for most of their children's plight; they appreciate the benefits of ART and, therefore, will take every step to motivate their children to adhere to medication.

The most common reason given by respondents for ART adherence in this study was “not falling sick as before.” This is evidenced by reduced hospital visits and admissions, improved patient's weight, and not missing schools like in the past prior to commencing treatment. Similar reason has also been reported by other studies [11, 12]. Other factors which enhanced adherence included provision of appropriate information and nutritional support which in our centre was by way of provision of Plumpy nuts, and existence of an active patient driven support group has also been documented by other studies [17–19].

Stigmatization was the strongest factor for nonadherence in the present study. Because of the strong influence of stigmatization, health education and information should be increased and sustained through the use of social media, religious gatherings, and even town criers where available (personal suggestion). This can positively influence the community acceptance of the disease thereby demystifying the associated stigma and increasing ART adherence. The barrier to adherence in this study is in contrast to the drug exhaustion at home as the main reason for nonadherence in some earlier studies [11, 12]. The reason for the drug exhaustion at home was sharing with patient's ARV medication with other siblings [12]. Surprisingly, drug side effects were not a major barrier to effective ART adherence in the present study. This agrees with a study in Kampala [14] but differs from the study of Iroha et al. [11]. The reason for the observed difference is not known.

## 5. Conclusion

The adherence level in this study was good. Stigma was an important reason given by the patient for nonadherence. There is need for concerted effort in addressing this barrier to improve adherence and prevent emergence of drug resistance and treatment failure.

## Figures and Tables

**Table 1:** Sociodemographic characteristics of the children studied (*n* = 210).

Characteristics	All patients (*n* = 210)	Adherent patients (*n* = 191)	Nonadherent patients (*n* = 19)	*p* value
*Age (months)*				
<12	3 (1.4%)	3 (1.6%)	0 (0%)	0.110
12–59	59 (28.1%)	57 (29.8%)	2 (10.5%)	
60–119	77 (36.7%)	71 (37.2%)	6 (31.6%)	
120–180	71 (33.8%)	60 (31.4%)	11 (57.9%)	
*Gender*				
Male	108 (51.4%)	97 (50.8%)	11 (57.9%)	0.750
Female	102 (48.6%)	94 (49.2%)	8 (42.1% )	
*Educational level*				
Not of school age	65 (31.0%)	64 (33.5%)	1 (5.3%)	0.004^*∗*^
Primary	85 (40.5%)	78 (40.8%)	7 (36.8%)	
Secondary	60 (28.5%)	49 (25.7%)	11 (57.9%)	
*Treatment duration (months)*				
<12	12 (5.7%)	12 (6.3%)	0 (0%)	0.001^*∗*^
12–35	106 (50.5%)	103 (53.9%)	3 (15.8%)	
36–59	68 (32.4%)	61 (31.9%)	7 (36.8%)	
>60	24 (11.4%)	15 (7.9%)	9 (47.4%)	
*Who administers the medication*				
Self (age ≥ 10 years)	26 (12.4%)	19 (9.9%)	7 (36.8%)	0.005^*∗*^
Biological parent	146 (69.5%)	138 (72.3%)	8 (42.1%)	
Biological grandparent	31 (14.8%)	28 (14.7%)	3 (15.8%)	
Others	7 (3.3%)	6 (3.1%)	1 (5.3%)	
*Orphan status*				
Both parents alive	124 (59.0%)	117 (61.3%)	7 (36.8%)	0.001^*∗*^
One parent alive	64 (30.5%)	60 (31.4%)	4 (21.1%)	
Both parents dead	22 (10.5%)	14 (7.3%)	8 (42.1%)	

^*∗*^Statistically significant.

**Table 2:** Reasons given by patients/caregivers for good adherence.

Reasons for adherence	Number^*∗*^ (*n* = 191)^*∗*^	Percent^*∗*^
No longer as sick as before	178	(93%)
Caregiver had good information	143	(75%)
Availability of nutritional support	70	(37%)
Seeks to stay alive	46	(24%)
Influence of support group in the centre	36	(19%)

^*∗*^Some respondents gave multiple reasons.

**Table 3:** Reasons given by patients/caregivers for poor adherence.

Reasons for poor adherence	Number^*∗*^ (*n* = 19)	Percent^*∗*^
Stigma	16	(84%)
Forgetfulness	6	(32%)
Caregiver not well	5	(26%)
Did not know drugs were daily	4	(21%)
Away from home	4	(21%)
Patient was sick	4	(21%)
Feeling healthy	3	(16%)
Unpleasant drug side effects	2	(11%)
Nonavailability of food	1	(5%)

^*∗*^Some respondents gave multiple reasons. Subjects/caregivers who gave more reasons have lower adherence to medications.
